# Ibuprofen-induced Anaphylactic Shock in Adult Saudi Patient

**DOI:** 10.7759/cureus.6425

**Published:** 2019-12-20

**Authors:** Talal A Shaikhain, Faisal Al-Husayni, Kareem Elder

**Affiliations:** 1 Internal Medicine, National Guard Hospital, Jeddah, SAU

**Keywords:** ibuprofen, allergy, anaphylaxis, anaphylactic shock, nsaids

## Abstract

Non-steroidal anti-inflammatory drugs (NSAIDs) are one of the most prescribed medications globally. They act through inhibiting cyclooxygenase (COX)-1 and COX-2 enzymes. In contrast to other NSAIDs, anaphylaxis due to ibuprofen is quite rare, especially in adults. The management of anaphylaxis depends on early recognition of the symptoms, administering epinephrine, and avoidance of the causing allergen. Here, we report a case of a 23-year-old female who presented with anaphylactic shock after ingesting ibuprofen.

## Introduction

One of the most prescribed medications are non-steroidal anti-inflammatory drugs (NSAIDs); it has been reported that NSAIDs are the second most frequent cause of drug-induced hypersensitivity after β-lactam antibiotics [1]. Furthermore, recent studies have shown NSAIDs causing frequent hypersensitivity reactions [2-3]. The estimated prevalence of NSAIDs hypersensitivity varies between 25% to 30% among patients with underlying diseases such as asthma and chronic urticaria, while in general population, it ranges between 0.1% and 0.3% [4-6]. Therefore, physicians are encouraged to recognize and appropriately manage such patients. NSAIDs’ mechanism of action has been studied widely. Mainly, NSAIDs function through inhibiting cyclooxygenase (COX)-1 and COX-2 enzymes which reduces prostaglandin production [7]. The outcome is a higher production of cysteinyl leukotrienes.

Hypersensitivity reactions are divided into non-immunological and immunological mediated reactions, and the latter is further divided into IgE or non-IgE mediated [8]. The exact mechanism of an allergic reaction to NSAIDs is not conclusive. However, the excess of prostaglandin production is more likely to be the cause [9].

Anaphylaxis and anaphylactic shock have been rarely reported to be caused by NSAIDs such as ibuprofen which is well-known and used worldwide. There are very few cases of ibuprofen-induced anaphylaxis and anaphylactic shock, and they are mainly reported in the pediatric population [10-11]. A possible explanation is that ibuprofen is commonly used in almost all age groups, and the allergic presentation would manifest at a younger age. Here, we report a case of an adult Saudi female who presented with a severe anaphylactic reaction after ibuprofen ingestion.

## Case presentation

**Figure 1:**
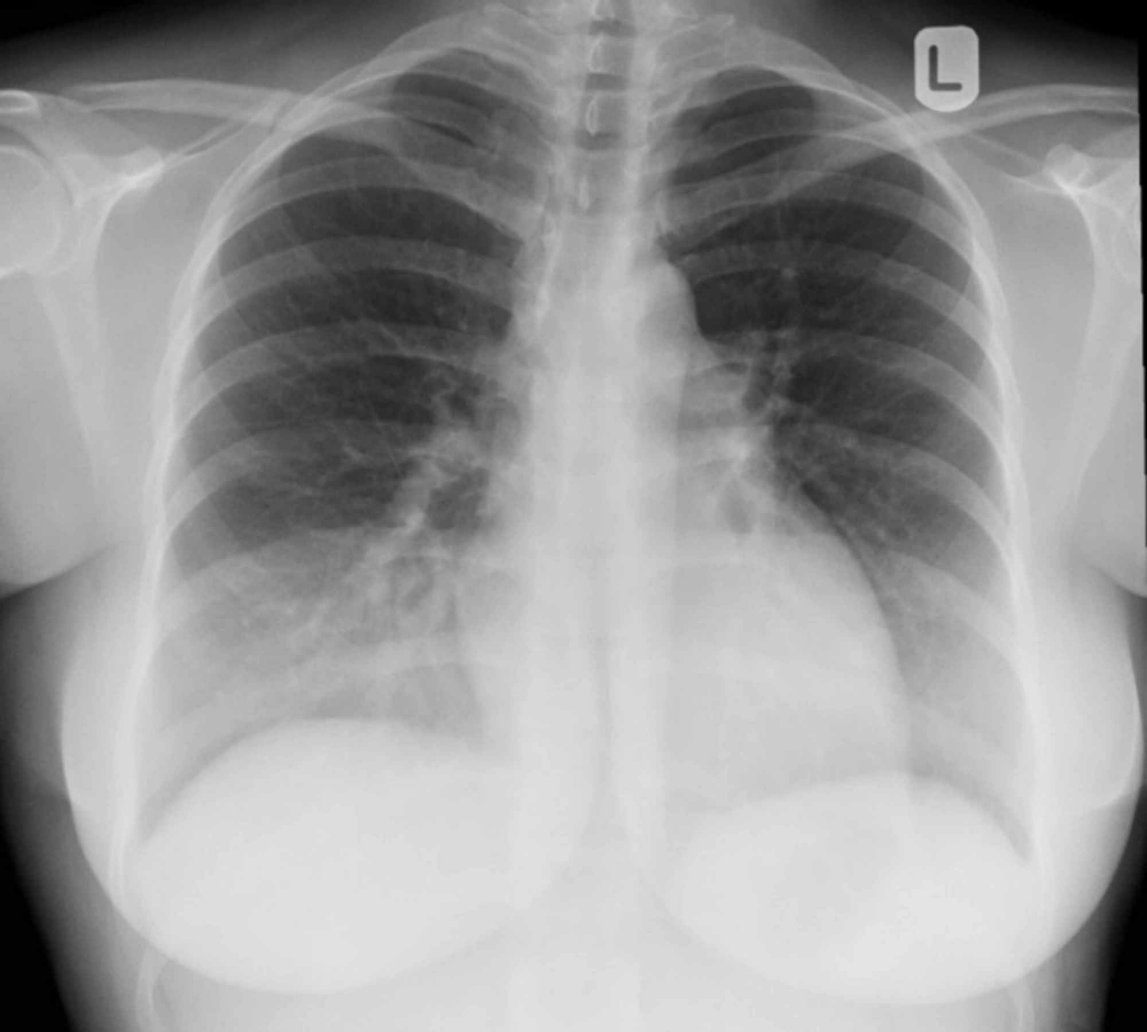
Patient’s chest X-ray showing normal appearance

A 23-year-old female with a history of hypothyroidism on 100 mcg of L-thyroxine, and not taking any other medication, presented to the emergency department with anaphylactic shock. The patient denied a personal or family history of food or substance allergy, hyperactive airway disease, atopy, or allergic rhinitis. Three days before her presentation, the patient was discharged after a spontaneous vaginal delivery of her first child. The patient was prescribed acetaminophen and ibuprofen to control the pain and to be taken as needed. On the third day after discharge, the patient experienced perineal pain that was not relieved by ingesting acetaminophen. The patient then took 200 mg of Ibuprofen as instructed, which she never had before. Five hours later, the patient started to experience itchiness all over her body associated with facial and lip swelling. On the way to the hospital, the patient started to have skin rash with throat and chest tightness. At presentation to the emergency department, the patient was drowsy with blood pressure of 72/43 and heart rate of 128. The patient had stridor, decreased breath sounds bilaterally, and urticaria. Intramuscular epinephrine (0.5 mg) was administered alongside with hydrocortisone (100 mg) and diphenhydramine (50 mg). The patient’s condition immediately stabilized, and her vitals normalized apart from epinephrine-induced tachycardia. Blood tests showed leukocytosis of 13.5K with a neutrophil count of 10.0K and a platelet of 872K. Chest X-ray was done and was normal (Figure 1). The patient was kept for 24-hour observation and then discharged with epinephrine auto-injector to be used when needed, along with prednisolone (20 mg) and loratadine (10 mg) daily for three days. She was also advised to avoid using ibuprofen and other NSAIDs. A month afterward, the patient did not have any similar episodes nor allergic reactions.

## Discussion

Recently NSAIDs were classified as the most common cause of hypersensitivity drug reaction overtaking β-lactam antibiotics [12]. NSAIDs are implicated in a wide range of adverse reactions ranging from simple rhinitis/rhino-sinusitis to anaphylactic shock and systemic vasculitis [13]. Classification of these reactions proved difficult due to multiple mechanisms and a variety of presentations. Adverse reactions can be non-immunological mediated by their action on COX-1 involved in the pathway of leukotrienes and prostaglandins, which can lead to different respiratory and cutaneous manifestations [8]. Immunological reactions can be mediated through IgE, T-cells and other pathways. The European Academy of Allergy and Clinical Immunology (EAACI)/European Network for Drug Allergy (ENDA) group classification is widely used and aims to categorize the different presentations and mechanisms. Five categories currently used are NSAID-exacerbated respiratory disease (NERD), NSAID-exacerbated cutaneous disease (NECD), NSAID-induced urticaria/angioedema (NIUA), single NSAID-induced urticaria/angioedema and anaphylaxis (SNIUAA), and lastly, single‐NSAID‐induced delayed reactions (SNIDR) [14-15]. Our patient’s anaphylaxis presentation is most consistent with SNIUAA. In SNIUAA, the mechanism is believed to be immunologic IgE mediated. It is a type I immediate hypersensitivity reaction, and there is no cross-reactivity with other groups of NSAIDs. However, there are reports of cross-reactivity among closely related NSAIDs [13,15-17]. In our patient's case, she had ibuprofen-induced anaphylaxis. Ibuprofen, in particular, is not commonly associated with anaphylaxis when compared to other NSAIDs [18]. Most of the data regarding ibuprofen anaphylaxis come from case reports and case series. A cohort of 41 patients reported having Ibuprofen hypersensitivity found only one patient to have anaphylaxis [19]. Another study regarding NSAIDs allergy revealed eight of 48 patients with ibuprofen allergy had anaphylaxis [20]. Our patient did not report any history of allergy to ibuprofen or other substances. Anaphylaxis is a clinical diagnosis. Serum tryptase can aid in the diagnosis of unclear cases. Serum tryptase requires early collection (1-3 hours after anaphylaxis) and can be challenging to obtain on time with the acuity of presentation and emphasis on emergency management once anaphylaxis is recognized. Also, a repeated sample after 24 hours of symptoms resolution may be helpful to diagnose anaphylaxis [20]. Drug specific IgE is available, but it is not recommended for diagnosis [15]. An essential consideration in the differential diagnosis is the possibility of NIUA rather than SNIUAA in this patient. Findings supporting NIUA rather the SNIUAA would include the presence of previous cutaneous reaction or exacerbation of previous cutaneous condition, and reaction to more than one type of NSAIDs. A second method that can be used in unclear cases is challenging with aspirin, and if a positive reaction occurred, a diagnosis of NIUA is likely. Clinically this distinction is important to make for few reasons as SNIUAA is associated with severe and possibly fatal reaction compared to NIUA, and cross-reactivity is not typically seen with SNIUAA; thus, patients might be able to use other NSAIDs or aspirin if needed; however, cross-reactivity is still possible.

## Conclusions

Ibuprofen has been used worldwide for many years. Despite the rarity of causing anaphylaxis, physicians and patients must be aware of the possibility of ibuprofen-induced anaphylaxis. Early recognition of the symptoms prevents probable devastating outcomes. Caution with other NSAIDs is warranted in patients with ibuprofen-induced anaphylaxis. The cornerstone management in any anaphylaxis case is avoiding the causing agent. Epinephrine auto-injector is a must carry-on for patients with life-threatening allergic reactions, and the importance of proper instructions of when and how to use it cannot be overemphasized.
